# Classifying Adenomyosis: Progress and Challenges

**DOI:** 10.3390/ijerph182312386

**Published:** 2021-11-25

**Authors:** Marwan Habiba, Giuseppe Benagiano

**Affiliations:** 1 Department of Health Sciences, University of Leicester, Leicester LE1 7HA, Leicestershire, UK; 2 Women and Perinatal Services, Leicester Royal Infirmary, Leicester LE1 5WW, Leicestershire, UK; 3 Department of Maternal & Child Health, Gynecology and Urology, “Sapienza” University of Rome, 00100 Rome, Italy; Giuseppe.benagiano@uniroma1.it

**Keywords:** adenomyosis, classification, endometriosis, fibroids, outer myometrium, inner myometrium, junctional zone, histopathology, MRI, ultrasound

## Abstract

Classically, the diagnosis of adenomyosis relied on histological examination of uteri following hysterectomy and classifications focused on the depth of endometrial invasion within the myometrium. There remain uncertainties around the cut-off point for the histological diagnosis. Imaging-based diagnosis enables recognition of the condition in women not undergoing surgery and facilitates the assessment of the extent of adenomyosis within the whole uterus, as well as of affections of the uterovesical pouch and of the pouch of Douglas. In this article, we explore the diagnostic uncertainties, the need to produce a classification of the condition and the challenges towards that goal. A distinction should be drawn between disease mapping and a classification that may link histological or image-based features with clinical characteristics, or with pathophysiology. An agreed system for reporting adenomyotic lesions may enable comparisons of research studies and thus contribute towards an informed classification. To this aim, we outline the features of the condition and explore the characteristics that are considered when producing a taxonomy. These include the latest proposal for subdivision of adenomyosis into an internal and an external variant. We also explore the uncertainties linked to classifying involvement of the uterovesical pouch, the pouch of Douglas and lesions in the outer myometrium. The limitations of currently available evidence suggest that agreement on a hypothesis to underpin a classification is unlikely at present. Therefore, current efforts will probably remain focused on disease mapping.

## 1. Introduction

In contrast to the case of endometriosis, public health professionals and the public at large are less aware of the consequences of uterine adenomyosis. Classically, adenomyosis is defined by the presence or ‘invasion’ of endometrial glands and stroma within the uterine muscle. Relevant features are the depth of stromal and glandular invasion and the presence of myometrial hypertrophy or hyperplasia leading to uterine enlargement. The reported incidence in different studies varies from 5 to 70%. However, this wide variability is more likely to be related to differences in diagnostic criteria, study population or methodological differences in case ascertainment [1]. Evidence exists that links adenomyosis to abnormal bleeding, painful menstruation, chronic pelvic pain, infertility, and spontaneous pregnancy loss but the health economic impact has not been clearly characterized. Adenomyosis is a very common finding in uteri removed by hysterectomy either as an isolated diagnosis or in conjunction with other conditions such as fibroids.

Both adenomyosis and endometriosis are due to the presence of endometrial tissue at ectopic sites and can result in heavy menstrual bleeding, pelvic pain, and infertility. Adenomyosis is highly prevalent in women undergoing hysterectomy and in women seeking fertility treatment [2,3]. This suggests considerable public health impact. Yet, this impact remains difficult to quantify and is rarely acknowledged. In turn, it has attracted insufficient research funding.

The lack of awareness may be related to difficulty in obtaining a reliable diagnosis. Until recently, diagnosis was only possible through histological examination following hysterectomy. Ultrasound and MRI are now widely available and increasingly used in clinical practice. These advances enabled non-operative assessment and renewed interest in adenomyosis and raised the prospect of better understanding of its etiology and impact [4,5]. Imaging-based diagnosing in women who are not undergoing hysterectomy brings into focus the limitations of currently availably therapeutic options and has raised the possibility that adenomyosis is a spectrum of diseases. The prevalence of adenomyosis in various populations is unknown and our knowledge of early stages of the disease remains limited.

One manifestation of the renewed focus on adenomyosis is the interest in developing a classification that has broad support. It is hoped that this will enable better appreciation of the wide health impact and comparison of the outcomes of conservative treatment options.

Many challenges remain towards realizing a satisfactory classification [6]. A major issue is the disagreement on the definition of adenomyosis when using histology as a gold standard [7]. A uniformly agreed reporting system may be the first step towards a classification that takes symptoms into account [8]. Imaging has shown an acceptable level of accuracy but the diagnostic precision for individual features is less clear [9] and, despite advances, the necessary skill and expertise is not universally available. Added to this is the complexity posed by other uterine pathologies that are often associated with adenomyosis.

In this article, we present a brief overview of the features of adenomyosis that are relevant to arriving at a classification and provide an analysis of previous and more recent attempts at producing a taxonomy. We refer to the proposed subdivision of adenomyosis into an internal and external variant and explore the uncertainties linked to classifying involvement of the utero-vesical pouch, the pouch of Douglas and lesions in the outer myometrium.

## 2. Epidemiology

The incidence of adenomyosis in the population is unknown. Adenomyosis is identified in 10–70% of hysterectomy specimens [10,11,12,13] and was diagnosed using ultrasound in 20.9% (95% CI: 18.5–23.6) of women attending a general gynecology clinic in a teaching hospital [14]. However, the high incidence in these selected populations does not provide an indication of the population incidence. Morassutto et al. [15] examined records of women attending health care facilities in the Friuli Venezia Giulia region of Italy. Based on histological diagnosis following hysterectomy, the incidence of adenomyosis in women aged 15–50 years was 0.027% and the calculated population prevalence was 0.17%.

Yu et al. [16] reported on the incidence of adenomyosis among women enrolled in the Kaiser Permanente Health Plan in the State of Washington. The cohort of 333,693 women contributed 1,185,855 woman-years. The diagnosis of adenomyosis was obtained based on diagnostic coding of health episodes. Most instances were confirmed following hysterectomy. The overall incidence of adenomyosis was 1.03% (28.9 per 10,000 woman-years). However, the estimates varied from 30.6 in 2007 to a low of 24.4 per 10,000 woman-years in 2014. The difference may be related to difference in hysterectomy rates. There was an age-related trend. The highest incidence was in the 41–45 years group; 18.0% also had endometriosis and 47.6% had uterine fibroids. Adenomyosis was higher in non-Hispanic black women and less common in Asian women. The health care burden was substantial: 82.0% of women had hysterectomies, nearly 70% had imaging studies suggestive of adenomyosis and 37.6% used chronic pain medications.

Menorrhagia or abnormal uterine bleeding, dysmenorrhea or pelvic pain, dyspareunia and infertility were reported in 90.8% of affected women. None of these symptoms is unique to adenomyosis. Adenomyosis often co-exists with endometriosis and fibroids which share the common symptoms. The specificity of preoperative diagnosis based on clinical features is poor and the role of imaging is increasingly being recognized.

## 3. Defining Adenomyosis

In 1925, Frankl proposed the term ‘adenomyosis’ to distinguish the condition [17]. Since then, there have been numerous attempts to reach a consensus on how to define and classify adenomyosis.

### 3.1. Histopathology

**Figure 1:**
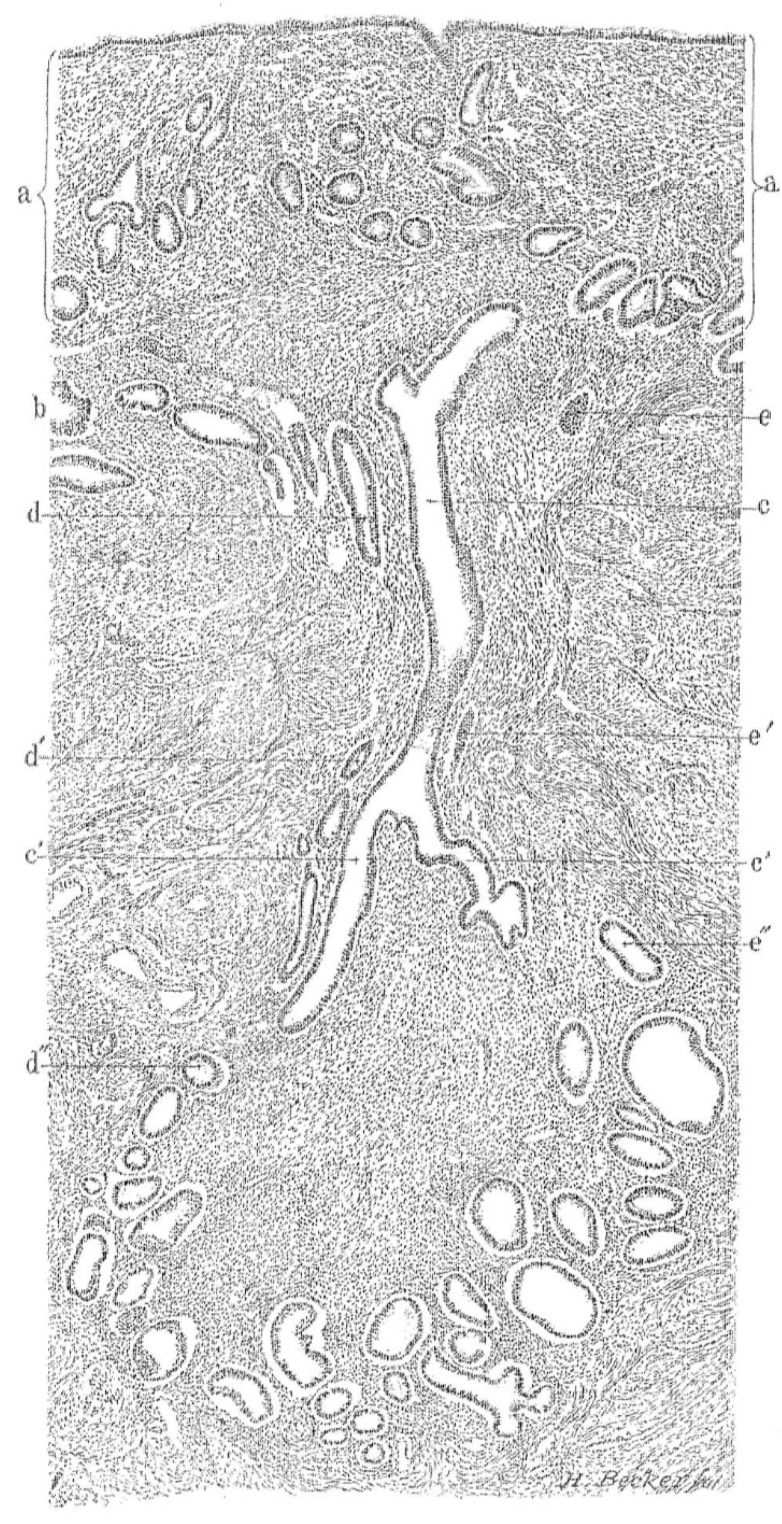
Histological section of the uterus with adenomyosis. The gland (c) within the muscle (b) is in continuity with the mucosa (a). The section also demonstrates gland continuity and convolutions deep within the muscle layer. In this illustration (c,c’) represent one gland and its branches and (d,d’,d’’) and (e,e’,e’’) represent adjacent glands. The illustration demonstrates the meticulous work by Thomas Cullen (1908).

**Figure 2:**
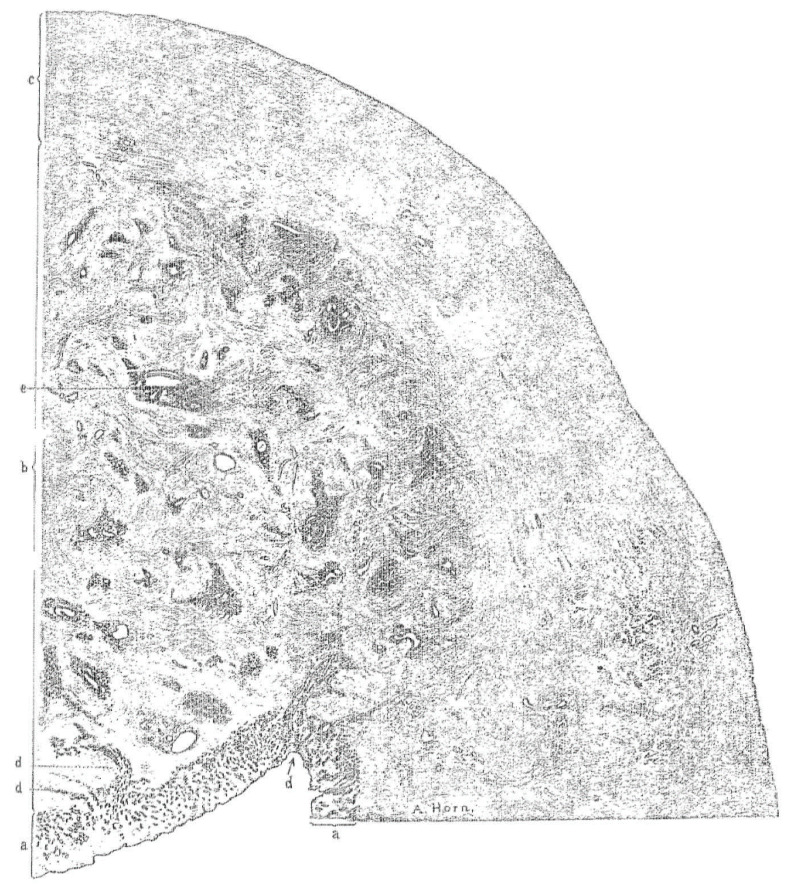
Section of the upper half of the posterior wall of the uterus with adenomyosis. The wall has three distinct zones: (a) Inner mucosa, (b) region with adenomyosis, (c) outer normal muscle tissue. The gland at (d) extends directly into the muscle and at (e) the gland is retracted from the surrounding stroma. The illustration demonstrates the meticulous work by Thomas Cullen (1908).

Early definitions focused on the presence of endometrial tissue within the myometrium in hysterectomy specimens (Figure 1) [18]. According to Lockyer [19], the first detailed description of the lesion we today identify as adenomyosis was made by Babeș (Victor Babesius) who presented a case of an intramural ‘myoma’ containing cysts lined with ‘low cubical epithelium derived from embryonic germs’ in 1882 [20]. Oskar Frankl [17] distinguished mucosal invasion within the myometrium and used the term ‘adenomyosis uteri’. Bird [10] defined adenomyosis as “the benign invasion of endometrium into the myometrium, producing a diffusely enlarged uterus which microscopically exhibits ectopic non-neoplastic, endometrial glands and stroma surrounded by the hypertrophic and hyperplastic myometrium”. This definition remains widely accepted. The classic diagnosis of adenomyosis relied on the identification of heterotopic endometrial glands and stroma within the myometrium. Myometrial hypertrophy, although itself difficult to define, is often viewed as relevant to gross or microscopic identification [21]. Whilst most discussions about adenomyosis and its diagnosis focus on the more common diffuse form (Figure 2), less commonly, adenomyosis can feature as a localized lesion within the myometrium (localized or discrete from).

The distinction between normal and adenomyosis has been debated over the years. Early writings viewed the condition as very rare and included descriptions of grossly enlarged and distorted uteri, but this was followed by a rapid rise in reported cases accompanied by calls for more strict diagnostic criteria and warnings against over-diagnosis [22].

One important aspect at the core of the debate is the almost universal irregularity at the endometrial myometrial interface. This poses a challenge in defining the cut-off point of what is normal. The notion of endometrial invasion refers to the vertical depth of endometrium presence below the mucosa and into the myometrium. The second significant challenge stems from the often-patchy distribution of adenomyosis within the myometrium. This means that the frequency of diagnosis can be related to the number of histological sections examined. This can be labor intensive and, as such, may not be pursued in routine clinical practice as it has no prognostic value once the uterus is removed [10].

As will be discussed below, definitions will remain subjective, unless identified features can be linked to specific clinical manifestations. This is challenging because the symptoms of adenomyosis are not pathognomonic. The uncertainty is reflected in the wide variation in reported cases of adenomyosis in hysterectomy specimens. This can be as high as nine-fold between reporting histopathologists [13]. It is also important to recognize that symptoms may not correlate with the depth of invasion or the extent of disease. Minimal disease or ‘adenomyosis sub-basalis’ which may be excluded by definitions that adopt a more conservative cut-off point, has been linked to symptoms [10,23]. This creates additional complexity in research in adenomyosis as it raises the question of the appropriateness of both the study and the chosen control groups. Most published literature adopted the customs of the local histopathology laboratory as a reference point (Table 1). These included the depth of microscopic high or low power fields, as a proportion of the myometrial thickness or, rarely, in terms of millimeters below the endometrial myometrial junction [22]. Given the high degree of uncertainty when applying the ‘gold standard’ definition, it is perhaps surprising that some of the studies that employed image-based diagnosis have claimed a high degree of accuracy. Myometrial hypertrophy that can account for uterine symmetrical enlargement linked to adenomyosis provided an additional feature to the clinical impression.

There is little research on the correlation between symptoms and the depth or extent of adenomyosis. The little evidence that is currently available suggests that dysmenorrhea is linked to the depth and extent of myometrial involvement, but that heavy periods are more common in women with less invasive disease [10]. All of this adds weight to the argument that histopathology should aim to report the actual depth of gland involvement rather than the presence or absence of disease. Reliance on histology meant that adenomyosis could, till recently, be only diagnosed in uteri removed surgically, most often in women who are symptomatic. Cullen referred to menstrual abnormalities and pain as the ‘expected’ symptoms and went on to state that the diagnosis could be readily made clinically including by his assistants [24]. Infertility is now recognized to be linked to adenomyosis.

### 3.2. Imaging

**Figure 3:**
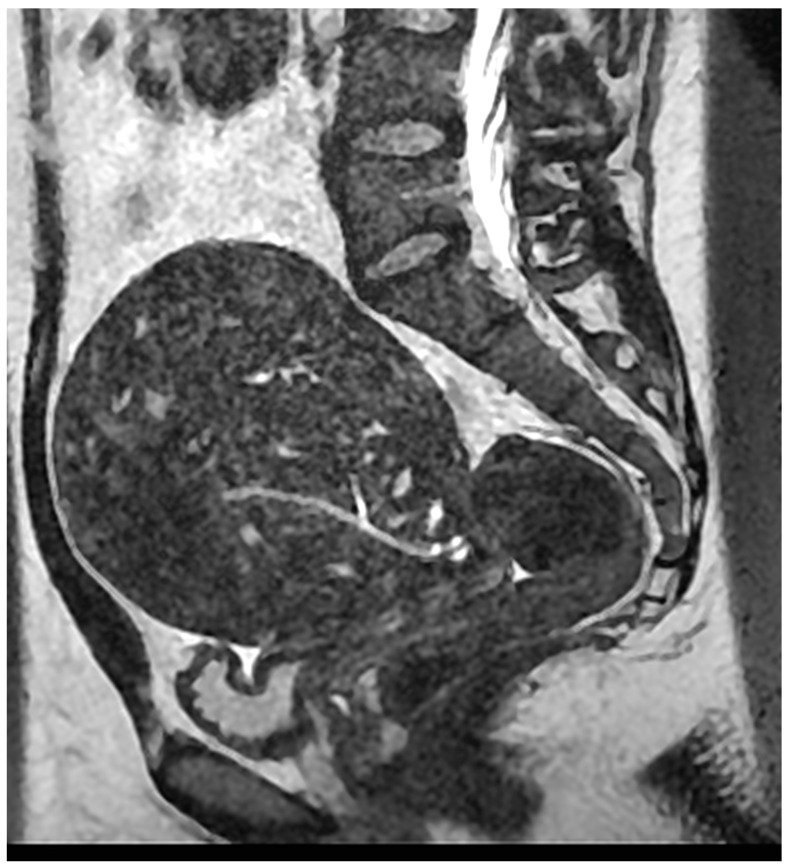
Magnetic resonance imaging (MRI) sagittal T2-weighted MRI showing a globular uterus containing multiple endometrial foci related to deep diffuse internal adenomyosis. Reproduced with permission from Habiba et al. (2020).

**Figure 4:**
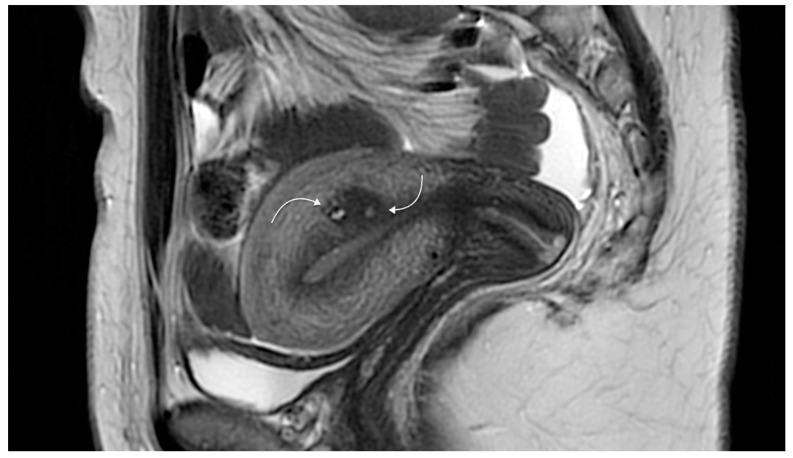
Magnetic resonance imaging showing sagittal two-dimensional T2-weighted magnetic resonance images showing a hypointense lesion containing tiny spots (curved arrows) and located in the posterior wall, adjacent to the endometrial cavity related to focal adenomyosis. Reproduced with permission from Habiba et al. (2020).

A new dimension to the diagnosis of adenomyosis came with the introduction of ultrasound and magnetic resonance imaging (MRI) (Figure 3 and Figure 4). Early efforts date back to the use of Gray Scale ultrasonography [25]. Hricak et al. [26] demonstrated the specific appearance on MRI of inner myometrial junctional zone (JZ), positioned between the endometrium and the outer myometrium, but did not link this to adenomyosis. Lee et al. [27] reported the identification of adenomyosis using MRI in hysterectomy specimens. Mark et al. [4] demonstrated the use of MRI in differentiating adenomyosis from fibroids. It has been pointed out that histological diagnosis relies on the detection of the ectopic glandular elements within the myometrium (the ‘adenosis’ component), whereas imaging relies on aberration in the appearance of the muscle (the ‘myosis’ component).

Non-invasive diagnosis offers an opportunity for the study of the natural history of adenomyosis, but efforts in this field will necessarily be hampered because of the limitation of resolution and because of the lack of agreement on the histological diagnostic ‘gold standard.’ The recognition of increased myometrial JZ thickness as a marker of adenomyosis provided an important clue to diagnosis. However, in line with similar features, the cut-off point for diagnosis will necessarily be rooted on a balanced probability of detection.

(a) Magnetic resonance imaging (MRI)

MRI showed promise for the non-invasive classification and interest in its use has grown over the last three decades. Critical in MRI diagnosis is the thickness and appearance of the JZ. JZ thickness >12 mm has been considered as indicative of adenomyosis [28], but this cut-off point is not universally agreed. Additional features that can aid the diagnosis include diffuse, low-intensity areas accompanied by tiny high-intensity spots seen subjacent to the endometrium [29]. Increased JZ thickness is used as an indirect indicator of adenomyosis. However, the genesis of this differential density is uncertain and the transition from the inner to the outer myometrium has been shown to be gradual with no distinct transitional point [30]. This may explain the lack of agreement on a cut-off point for the diagnosis [31].

Sensitivity and specificity of MRI seem satisfactory, but interpreting images needs to consider a number of variables including age, phase of the menstrual cycle, gravidity, parity, hormonal status, previous uterine surgery and uterine contractions. In addition, the JZ is not measurable in 20–30% of women [32].

(b) Ultrasound imaging

Features of adenomyosis were reported using transabdominal ultrasound (TAS) [33,34], following the first report by Walsh et al. [25]. However, it was transvaginal scanning (TVS) that enabled better characterization [35,36] and proved to be more reliable [37]. TVS was also reported to be accurate for the diagnosis of leiomyoma and for combined adenomyosis and leiomyoma, but not to be specific for the diagnosis of adenomyosis only when compared to histopathology [38].

Brosens et al. [37] reported that ill-defined myometrial heterogeneity is a better predictor of adenomyosis compared to uterine morphometry. Further advances enabled more detailed characterization of the diagnostic criteria [39,40]. The diagnostic features of adenomyosis on ultrasound include: A globally enlarged uterus, a symmetrically enlarged uterus; cystic myometrial lesions surrounded by a hyperechoic halo; inhomogeneous, irregular myometrial echo texture, indistinct areas with increased or reduced echogenicity; subendometrial lines and buds, myometrial hypoechoic linear striations radiating with thin acoustic shadows not arising from echogenic foci or leiomyoma; indistinct endometrial–myometrial border, diffuse minimal vascularity within the myometrium; the question mark sign or a retroflexed uterus where the cervix is directed forward.

The probability of adenomyosis in a woman with heavy bleeding and positive ultrasound features is 68.1% and the probability of adenomyosis after a normal transvaginal ultrasound scan is 10%. The sensitivity and specificity for symptomatic women are 84.3% and 82.3%, and for all women undergoing hysterectomy are 81.1% and 85.1% [41].

A further development has been the introduction of three-dimensional (3D) ultrasound. Features linked to adenomyosis on 3D-TVS are: (i) Maximum Junctional Zone thickness (JZmax) ≥ 8 mm; (ii) myometrial asymmetry; (iii) hypoechoic myometrial striations. Exacoustos et al. [42] compared features of adenomyosis detectable on two- (2D) and three-dimensional (3D) TVS and correlated these with histopathologic features in the JZ and the outer myometrium. The presence of myometrial cysts was the most specific 2D-TVS, and heterogeneous myometrium was the most sensitive feature. The 3D-TVS markers linked to high sensitivity and the best accuracy were a JZ difference ≥ 4 mm and JZ infiltration and distortion. However, the metanalysis by Andres et al. [43] reported no improvement in overall accuracy using 3D-TVS compared with 2D-TVS.

Champaneria et al. [44] undertook a systematic review and meta-analysis of published articles that compared the diagnostic accuracy of TVS or MRI and that used histological diagnosis as the gold standard comparator. They analyzed three studies that reported on the use of MRI and six studies that reported on the use of TVS. The pooled sensitivity and specificity of TVS was 72% (95% CI: 65–79%) and 81% (95% CI: 77–85%), respectively. TVS had a positive likelihood ratio of 3.7 (95% CI: 2.1–6.4) and a negative likelihood ratio of 0.3 (95% CI: 0.1–0.5). The pooled sensitivity and specificity for MRI were 77% (95% CI: 67–85%) and 89% (95% CI: 84–92%) respectively. MRI had a positive likelihood ratio of 6.5 (95% CI: 4.5–9.3), and a negative likelihood ratio of 0.2 (95% CI: 0.1–0.4). Reported studies, however, used different cut-off points for histological diagnosis and included different cohorts of women which is reflected in the variations in the incidence of adenomyosis. Furthermore, little account is provided of the impact of concomitant pathology on the diagnosis [22].

The role of ultrasound is less clear when it comes to identifying the extent of adenomyosis. Sonography and histopathology concurred in only 57% of cases when assessing the depth of presence of endometrium within the myometrium and in only 23% of cases when assessing the degree of involvement (i.e., the volume of the uterine muscle tissue affected by the disease) and lesion density [45]. Available literature does not provide an indication of the diagnostic value of each of the above features and diagnostic accuracy remains operator dependent.

The Morphological Uterus Sonographic Assessment (MUSA) Group issued a consensus statement on terms, definitions, and measurements to be used when describing sonographic features of the myometrium [46]. It requires a detailed description of any lesions identified by ultrasound, not merely a positive or negative diagnosis. This involves description of disease location in the uterine wall affected (anterior, posterior, left lateral, right lateral, fundal); whether the lesion is focal or diffuse; determination of the presence of myometrial cysts; the degree of myometrial depth involvement (limited to the inner portion, invading the body of the uterus, reaching the serosa); the volume of the uterus affected (<25%, 25–50%, >50%); and size of the lesions [47]. This approach has considerable potential in enabling the understanding of symptoms and outcomes of treatment. However, how widely it will be adopted remains to be seen. As mentioned above, reliable imaging-based diagnosis is not widely available and inter-observer reproducibility remains a challenge. Several additional factors, such as patient’s age, phase of the menstrual cycle, parity, previous uterine surgery or associated pathology and uterine contractions can affect ultrasound features.

## 4. Variant Forms Related to Adenomyosis

(a) Uterine cystic adenomyosis

A rare variant that is encountered in adolescents and young adults is the uterine cystic form, which is characterized by the presence of a cystic lesion lined by endometrium within the myometrium. This may be present near the round ligament insertion into the uterus. Typically, it presents as early onset dysmenorrhea resistant to conservative management. The diagnosis can readily by made using imaging [48].

(b) Other rare variants

Several rare conditions which also comprise an admixture of endometrium and myometrium have been described. Their relation to adenomyosis and pathogenesis is debated. These lesions include [49]:

Uterus like Mass (ULM): Such a structure can be present in the pelvis and may be related to the ovary. Typically, it comprises a central cavity lined by endometriotic tissue surrounded by a thick wall of smooth muscle cells similar to the myometrium. Lesions can reach a large size. An ‘extrauterine adenomyoma’ can resemble a ULM but is characterized by the absence of a uterus-like structure and appears as a fibroid with scattered endometriotic foci.

Endomyometriosis: The term is used in literature to describe lesions similar to ULM and is preferred by those who regard these lesions as a variant of endometriosis with severe smooth muscle metaplasia.

Adenomyoma: The term was used during the second half of the XIX and the first three decades of the XX century, to refer to both adenomyosis and endometriosis [18]. Today, the term refers to the presence of a well-defined discrete adenomyotic nodule within the myometrium. A rare variety is the extrauterine adenomyoma that can arise in the ovary, the intestine, the omentum or elsewhere.

Polypoid adenomyoma and Adenomyomatous polyp: The terms, which may refer to the same entity, describe polypoid structures containing endometrium and myometrium.

Atypical polypoid adenomyoma or Adenomiofibroma: This variant is distinguished because the epithelial component exhibits atypia and possible squamous metaplasia.

These lesions are infrequently reported in literature. ULM, endomyometriosis and adenomyomatous polyp are mostly the subject of individual case reports. There are small case series of polypoid adenomyoma [50] and atypical polypoid adenomyoma [51].

## 5. Co-Existing Pathology

Adenomyosis often co-exists with other pelvic pathology, particularly endometriosis and uterine fibroids. In women undergoing hysterectomy, fibroids were present in 37% of cases with adenomyosis (*n* = 137) in the series by Weiss et al. [52], and in 32.9% of those with adenomyosis (*n* = 419) in the series by Shaikh and Khan [53]. The incidence was lower (22.8%) when the diagnosis was made by ultrasound in women attending a gynecologic clinic [14]. A higher incidence of fibroids in women diagnosed with adenomyosis (47.6%) was reported by Yu et al. [16]. A number of studies found a similar incidence of fibroids and endometriosis in women with and without adenomyosis, which raises the questions of whether the conditions are linked and of their relevance to symptoms [11,52,53].

### Relation between Adenomyosis and Endometriosis

It is well recognized that endometriosis and adenomyosis often co-exist, but the reported degree of association varies widely. Based on histological diagnosis, endometriosis was identified in 3% of cases of adenomyosis in the study by Weiss et al. [52] and in 4.6% in the study by Shaikh and Khan [53]. Using ultrasound, concomitant endometriosis was reported in 4.9% of cases [14]. The study by Yu et al. [16] reported concomitant endometriosis in 18% of cases. However, many of the studies that did not enroll highly selected populations and that contained a control group reported a similar incidence of endometriosis in the adenomyosis and the control groups [16]. This may suggest that the conditions are not necessarily linked [52].

In the work of Leyendecker and his group, using a selected population, adenomyosis was diagnosed based on MRI features of the junctional zone in 79% of women with endometrioisis, compared to 28% of women without endometriosis [54]. In another highly selected group, the prevalence of endometriosis in adenomyosis was 80.6% and the prevalence of adenomyosis in endometriosis was 91.1% [55].

In another cross-sectional study of a highly select group from a tertiary referral center, using surgical assessment and MRI-based diagnosis of adenomyosis, Chapron et al. [56] made a distinction between isolated ‘diffuse adenomyosis’ and ‘focal adenomyosis of the outer myometrium’ (FAOM). In this series of 175 uteri identified with adenomyosis, 30.2% consisted of isolated diffuse adenomyosis, 42.3% were FAOM and 27.4% were mixed. The study population was highly selected as evidenced by the high incidence of endometriosis which was present in 87.4% of those with (*n* = 175) and in 71.8% of those without adenomyosis (*n* = 117). Nevertheless, there was a significant increase in the frequency of FAOM in endometriosis-affected women compared to controls. The term ‘focal adenomyosis’ refers to adenomyotic foci located in the outer shell of the uterus that are separated from the JZ by healthy muscular tissue [56,57], but focal disease is not restricted to the outer myometrium and the term has also been applied to focal affections of the outer, middle and inner myometrium [58,59]. Larsen et al. [60] compared the incidence of adenomyosis in 153 patients with suspected deep infiltrating endometriosis (DIE, also referred to as rectovaginal endometriosis) and a reference group of 129 women. Adenomyosis was identified by MRI in 34.6% of the women with suspected DIE and in 19.4% of the control group [60].

Relevant to the ongoing debate about the relation between adenomyosis and endometriosis is the distinction between ‘internal’ and ‘external’ adenomyosis. The first is characterized by the presence on MRI of focal or multifocal intra-myometrial tiny cystic structures; the second is made up of lesions in the outer portion of the myometrium [59]. The external variant may thus arise from invasion of the myometrium by endometriotic lesions. The opposing view is that adenomyotic lesions travel through the myometrium, giving rise to DIE and bladder lesions [6].

Another dimension was added to the debate by Kishi et al. [61]. On the basis of the MRI, they distinguished four subtypes of adenomyosis: subtype I (intrinsic adenomyosis) involves lesions directly connected to the eutopic endometrium and is characterized by a thickened JZ; subtype II (extrinsic adenomyosis) refers to cases in which lesions are noted in the outer myometrium, and where the JZ seems to be intact on MRI; subtype III (intramural adenomyosis) is the variant in which foci are separated from the JZ and from the serosa; and subtype IV (indeterminate adenomyosis) includes cases that do not conform to any of the above criteria [61]. Differences in biomarker expression were taken to link DIE to extrinsic adenomyosis [62,63].

## 6. Pathophysiology of Adenomyosis

Detailed exploration of the pathophysiology of adenomyosis is beyond the scope of this article, but a brief description of major features will help in understanding the condition.

There is no basement membrane separating the endometrium from the underlying muscle layer. The inner myometrial layer immediately underlying the endometrium appears on MRI as a distinct area known as the Junctional Zone (JZ). Adenomyosis may arise directly as an abnormal ingrowth and invagination of the basal layer of the endometrium into the myometrium. Such process could be enhanced through disruption and repair, as may occur following surgical curettage or placental invasion. These can trigger a cascade of tissue injury and repair, leading to further disruption and invasion by the endometrium. The endometrium may ‘invade’ between muscle bundles or along lymphatics. Invasiveness could occur from the outer surface of the uterus either from endometriosis or from deeper affections of the pouch of Douglas. An alternative hypothesis is that the ectopic endometrium is derived from multipotential perivascular cells that acquired their location during embryogenesis. Epithelial–mesenchymal transition at the interface between the myometrium and the endometrium may play a role in the pathophysiology of adenomyosis. Adenomyosis has also been linked to aberrations of both the endometrial and the myometrial compartments [9,64,65,66].

## 7. Classifying Adenomyosis

Over the years, a number of proposals have been put forwards to classify adenomyosis (Table 2). Early efforts used histological criteria with a focus on mapping the depth of endometrial invasion within the myometrium, both to define the extent of the disease and to distinguish a cut-off point for diagnosis. An important driver was to avoid over-diagnosis. The distinction between adenomyosis and the normal irregularity at the interface between the endometrium and the myometrium remains a challenge. This is particularly the case because of the difficulty researchers encounter when attempting to establish a correlation between histological features and symptoms. It is perhaps not surprising that researchers have proposed different cut-off points. On the other hand, because hysterectomy is usually performed on symptomatic women, a stringent cut-off point risks under-diagnosis.

The introduction of imaging-based diagnosis enabled a global view of the uterus and provided a less labor-intensive opportunity for disease mapping compared to histological diagnosis. A distinction, however, needs to be made between disease mapping based on anatomical location of lesions and disease categorization rooted on symptom severity or prognostic indices. Each of these has its own challenges. Most published literature reports on the accuracy of diagnosis based on whether adenomyosis is present or absent, rather than on the accuracy of each individual feature, location or cut-off point for diagnosis. The severity of symptoms attributed to adenomyosis has not been shown to be predictable based on its topography within the myometrium. A contrary view was expressed by Weiss et al. [52], who argued that adenomyosis is a normal variant rather than a disease entity. This was based on their study of 137 patients who underwent a hysterectomy, including 48% with adenomyosis. Those with and without adenomyosis had the same prevalence of fibroids, endometriosis, abnormal bleeding and chronic pelvic pain [52]. The latter point is important, but not unique to adenomyosis. It is well recognized that the extent of endometriosis or the size of fibroids do not always correlate with symptom severity.

Another feature that has attracted debate with regards to the classification of adenomyosis is the relation between the ‘classic’ adenomyosis of the uterus and lesions in the pouch of Douglas and the uterovesical pouch. These exhibit histological similarities to adenomyosis. The debate about their pathogenesis and whether they originate from spread of uterine disease to surrounding tissues or spread of endometriosis into the myometrium has important implications when it comes to classification. Similarly, affections of the outer myometrium may represent invasion from endometriosis lesions implanted on the serosa. Understanding the pathophysiology can also have other implications for classification. Adenomyosis may represent a spectrum of diseases with the unifying feature of ectopic endometrium and stroma within the myometrium [57].

One consideration for classification of adenomyosis is the place of JZ hyperplasia and whether it is pathognomonic of adenomyosis. Rasmussen et al. [64] attempted to classify the disease when confined to the inner myometrial and JZ regions into three separate ultrasound-based categories: (i) Adenomyosis of the inner myometrium; (ii) junctional zone disease, characterized by a serrated appearance of the JZ; and (iii) linear junctional zone [64]. While the existence and importance of JZ hyperplasia is not uniformly agreed, it was defined as partial or diffuse thickening of the JZ from 8 mm and over to less than 12 mm in the absence of additional imaging signs of adenomyosis [9]. Some investigators suggested that adenomyosis could be divided into different categories, based on morphology and location of the lesion. The term ‘diffuse adenomyosis’ is applied to lesions that affect at least one myometrial wall and could be symmetric or asymmetric. A further subdivision into three categories according to the depth of involvement reaching less than one-third, less than two-thirds, greater than two-thirds of the myometrium has also been suggested [9]. This is distinct from ‘focal adenomyosis.’ Finally, an ‘adenomyoma’ is represented by a myometrial mass with indistinct margins of primarily low-signal intensity on T2-weighted MRI sequences [9,59,67] and it could be solid or cystic; it is commonly located in the mid-myometrium and rarely protrudes into the endometrial cavity or under the serosa [59]. A new variant, ‘external adenomyosis’ (anterior or posterior), has recently been introduced for lesions found adjacent to the uterine serosa, being significantly associated with pelvic endometriosis.

The International Endometrial Tumor Analysis (IETA) Group was established to classify the sonographic features of endometrial and intrauterine lesions [67]. The consensus criteria were given the acronym MUSA (Morphological Uterus Sonographic Assessment) [46]. The features proposed for adenomyosis are: the location of the disease (anterior, posterior, left lateral, right lateral, fundal); a classification of the lesions (focal or diffuse); the presence or absence of intra-lesion cysts; the involvement of the myometrium (limited to the inner portion, invading the body of the uterus, reaching the serosa); the extent of the disease (affecting <25%, 25–50%, >50% of the uterine volume); and the size of the lesions [47].

Recently, Exacoustos et al. [68] studied the relation between an ultrasound-based disease classification and symptoms. Women with ultrasound diagnosis of diffuse adenomyosis were older and had heavier menstrual bleeding compared to those with focal disease, but there were no statistically significant differences in the severity of dyspareunia and dysmenorrhea. Focal disease was associated with a higher percentage of infertility. Overall, there was no direct correlation between ultrasound depictions of the extent of the disease and symptoms [68]. The authors speculate that this may be related to co-existent pathology, or to a true lack of correlation between symptoms and disease extent. However, larger studies are needed before definitive conclusions can be reached.

## 8. Conclusions

Despite the significant advances in our understanding of adenomyosis, a satisfactory classification of the condition remains challenging because of the limitation of our understanding of the clinical correlates. A pathophysiology-based classification also remains illusionary. It is hoped that recent attempts at detailed disease mapping will provide the information needed towards a clinically useful classification.

## Figures and Tables

**Table 1:** The histological cut-off point adopted for the diagnosis of adenomyosis in different studies.

Reference	Descriptor
	Microscopic field of view
Novak and Woodruff, 1979	>1 high-power field.
Parazzini et al., 1997	>0.5 of low-power field
Zaloudek and Hendrickson, 2002	>0.5 of low-power field
Gompel and Silverberg, 1985	medium-power field (×100 lens)
Owolabi and Strickler, 1977	>low-power field
	Uterine wall thickness
Hendrickson and Kempson, 1980	>1/4 of the total thickness
Shaikh and Khan, 1990	>1/3 to 1/4 of the total thickness
	Measurement in mm
Bergholt et al., 2001	recommend 3 mm as cut-off
Levgur et al., 2000	≥2.5 mm

**Table 2:** The main features used in proposed classification systems for adenomyosis.

Reference	Categories/Basis of Classification
	**Histopathology**
Bird et al. (1972) Sammour et al. (2002)	Depth of invasion within the mometrium Degree of involvement: Number of glands per low power field
Siegler and Camilien (1994) Hulka et al. (2002) Vercellini et al. (2006)	Depth of penetration Degree of involvement Configuration: Diffuse, discrete.
Levgur et al. (2000)	Depth of invasion
	**Ultrasound based**
Lazzeri et al. (2018)	Configuration Depth of lesion (affecting Junctional zone, inner, outer myometrium)
	**MRI based**
Grodts et al. (2008)	JZ hyperplasia Adenomyosis Adenomyoma
Kishi et al. (2012)	Subtype I: Intrinsic: Inner uterine layer Subtype II: Extrinsic: Outer uterine layer (normal JZ) Subtype III: Solitary adenomyosis no connection to the JZ or to the serosa. Subtype IV: Indeterminate
Kobayashi and Matsubara (2020)	Depth of lesion Configuration Size of lesion Localization of lesion Concomitant pathology
Bazot and Daraï (2018)	A Internal adenomyosis Configuration B Adenomyomas Consistency C External adenomyosis Location Coexisting endometriosis
Grimbizis et al. (2014)	Configuration Polypoid forms Special (rare forms)
	**Features at surgery**
Pistofidis et al. (2014)	Consistency
	**Imaging based**
Gordt et al. (2018)	Extent Localization Configuration Consistency Size

## Data Availability

Not applicable.
